# The Effects of Morphine on Tissue Structure of the Growth Plate in Male Rats

**Published:** 2011

**Authors:** Massood Ezzatabadipour, Masoud Majidi, Reza Malekpour-afshar, Seyed Hasan Eftekharvaghefi, Seyed Noureddin Nematollahi-mahani

**Affiliations:** 1 *Neuroscience Research Centre, Kerman University of Medical Sciences, Kerman, Iran *; 2 *Department of Anatomical Sciences, Afzalipour School of Medicine, Kerman University of Medical Sciences, Kerman, Iran*; 3 *Department of Pathology, Afzalipour School of Medicine, Kerman University of Medical Sciences, Kerman, Iran*

**Keywords:** Cell proliferation, Growth plate, Morphine

## Abstract

**Objective(s):**

Studies have shown that morphine, in addition to its analgesic properties, has several effects on cell proliferation and apoptosis. There is also evidence that opioid receptors are present on chondrocytes. Our main objective in the present study was to investigate the effects of morphine on rat femur growth cartilage.

**Materials and Methods:**

This research was carried out on 18 4-week-old male rats. Animals were divided into four groups: groups 1 (n= 3) and 2 (n= 4) were non morphine-dependent and groups 3 (n= 6) and 4 (n= 5) were morphine-dependent. Groups 1 and 3 were followed up for 4 weeks and the others for 7 weeks. We prepared femur bone biopsies, fixed the samples in 10% formalin and 10% nitric acid and stained the samples with haematoxylin and eosin. The thickness of the growth cartilage and its proliferative zone (PZ) cell number were studied. In addition, the existence of necrosis, inflammation, fibrosis, and hyalinisation were evaluated.

**Results:**

There were no signs of inflammation, fibrosis, necrosis or hyalinisation in the growth cartilages of all rats. The morphine-dependent groups had a statistically significant difference (*P*< 0.001) in the number of cells in the proliferative zone and thickness of the growth cartilage compared to other groups using ANOVA analysis.

**Conclusion:**

It seems that morphine reduces the number of cell in the proliferative zone and decreases the thickness of the growth cartilage which may alter longitudinal growth of long bones.

## Introduction

Morphine, the main alkaloid of opium, in addition to its analgesic properties, has been shown to affect programmed cell death (apoptosis) and tissue necrosis (1). The results of studies on human joints have revealed the existence of μ-opioid receptors on cultured chondrocytes which affect CREB (cyclic AMP responsive element binding protein) gene expression (2). 

There are five morphologically distinct zones in growth plate cartilage from its expanded articular end to the diaphyseal centre: the reserve zone, the proliferation zone (PZ), the hypertrophic zone (HZ), the calcification zone and the ossification zone (3). Each zone shares differently in the development of longitudinal bone growth. For instance, considering the proximal growth cartilage of the tibia in a 4-week-old rat, 9% of growth is contributed by the proliferation zone, 32% by the calcification zone and 59% by the hypertrophic zone (4). In rats, the rate of longitudinal bone growth increases between one to five weeks and then declines between 11.5 to 13 weeks (5,6).

Various factors influence the proliferation and differentiation of chondrocytes and also change the longitudinal growth of bones. Longitudinal growth of bones is a dynamic process which is regulated by growth hormones (mediated by somatomedin produced in the liver) with important contributions from glucocorticoids and thyroid hormones. These factors stimulate cell proliferation via insulin-like growth factor-1 (IGF-1) and binding to specific receptors on chondrocytes (7). 

The results of some studies have shown that serum luteinising hormone (LH) and testosterone levels decrease following the administration of morphine (8). A study by Karimi-Mobarakah *et al* (2004) has shown that experimentally injured bones have significantly delayed healing after morphine administration (9). 

Although there are some reports in the literature dealing with various effects of morphine on different tissues, the probable effects of chronic morphine administration on growth plate cartilage structure has not been elucidated. We conducted the present research to study the effect of morphine on the morphology and cell population of femur growth plate cartilage and its thickness in male rats. 

## Materials and Methods

This study was conducted on eighteen 4-week-old male Sprague-Dawley rats. The animal house of Kerman Neuroscience Research Centre, Kerman, Iran, provided the rats for this study. An institutional review board approval (EC/KNRC/85-36) was obtained from Kerman University of Medical Sciences. Animals were maintained at 25±3 °C with a 12 hr light-dark cycle. All animals had free access to water and rodent chow.

 We randomly divided the animals into four groups. Non morphine-dependent animals were divided into the control group for four weeks of treatment (n= 3) and the control group for seven weeks of treatment (n= 4). Morphine-dependent animals received morphine in their drinking water for four weeks (n= 6) and seven weeks (n= 5). 

In order to make the animals morphine-dependent, we started with 0.1 mg/ml morphine at days one and two, and then increased it as follows: 0.2 mg/ml at days three and four, 0.3 mg/ml at days five and six and 0.4 mg/ml after day six (10). In order to mask the bitter taste of morphine, 50 g of sucrose was added to one litre of drinking water. To verify the development of the desired dependence on morphine, we tested two randomly-selected rats from the morphine-dependent animals in the four and seven week treatment groups. Animals received a subcutaneous injection of naloxone 1mg/kg (naloxone hydrochloride, 0.4 mg, Tolid daru, Iran) (11). Dependence on morphine was confirmed by the occurrence of withdrawal symptoms such as writhing, diarrhoea, wet shaking and jumping (12,13). After the desired follow-up period, the animals went under complete anaesthesia by intraperitoneal injection of chloral hydrate (400 mg/kg) (14). Then, the femurs were removed and fixed in 10% formalin in PBS solution for 48 hr followed by 10% nitric acid solution for 72 hr. Tissue processing, including dehydration in graded ethanol, clearing in xylene and embedding in paraffin was done using a tissue processor apparatus (Automatic Tissue Processor, Ds 2000/H, Did Sabz, Iran). We prepared 5 μm thick sections of the distal growth cartilage of the specimens and used haematoxylin and eosin staining for histopathological studies. The cutting operator and the pathologist were blind to the arrangements of the study. 

Using digitally captured 400× images from five similar zones of growth cartilages, we assessed the following parameters: cell density in the proliferative zone (PZ) as well as the existence of necrosis, inflammation, fibrosis, and hyalinisation. Two examiners carried out cell counts separately using Analysis^®^ software (Olympus, Japan). For measuring the thickness of the growth plate cartilage, we used a calibrated optical micrometer. 

Data are presented as mean±SEM. SPSS version 16 software was used for data analysis. Groups were compared by one-way analysis of variance (ANOVA) followed by Tukey’s *post-hoc* test. *P*< 0.05 was considered as significant. At first, the dependence of animals on morphine and then the duration of dependence (four and seven weeks) were considered.

## Results

**Figure 1.**
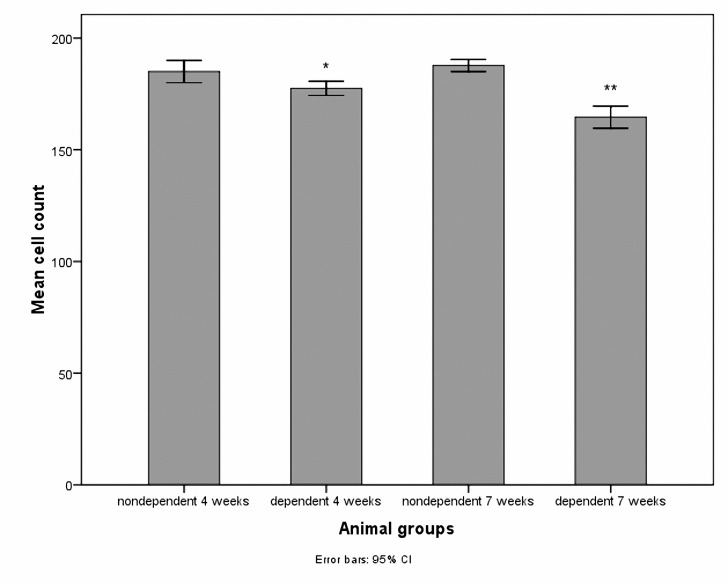
Bars are representative of the cell number in the proliferation zone in different groups. *P< 0.05 compared with non morphine-dependent 4 weeks. **P< 0.001 compared with non morphine-dependent 7 weeks.

**Figure 2.**
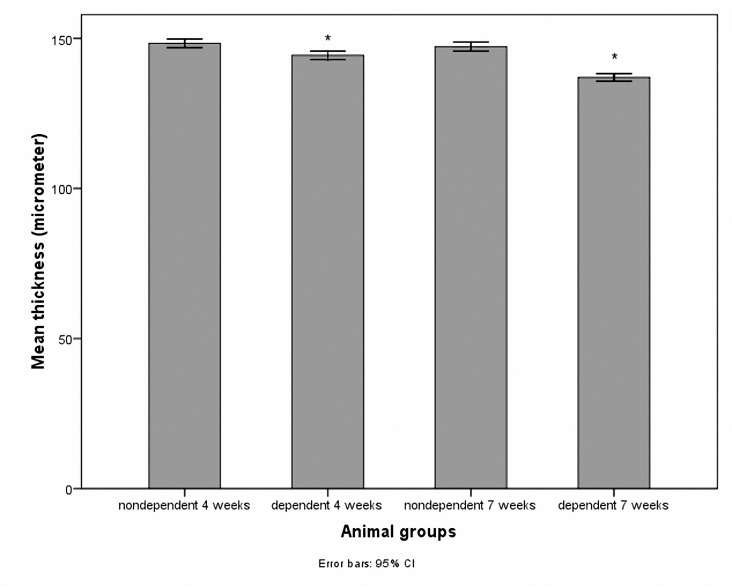
Bars are representative of the growth plate thickness in different groups. * *P*< 0.001 compared with the non morphine-dependent groups.

The mean cell numbers in the growth plate PZ is demonstrated in  Figure 1. Cell numbers were reduced in the morphine-dependent groups at both studied time points. The results of ANOVA regarding four and seven weeks duration of dependency (*P*< 0.001) are shown in  Table 1. The growth plate thickness decreased significantly (*P*< 0.001) in the morphine-dependent groups after both four and seven weeks of morphine administration (Figure 2). As the time passed, the number of cells in the PZ and the thickness of the growth plate decreased significantly in the morphine-dependent group, but neither the cell number in the PZ nor the growth plate thickness changed significantly in the non morphine-dependent group (Table 1). 

**Figure 3.**
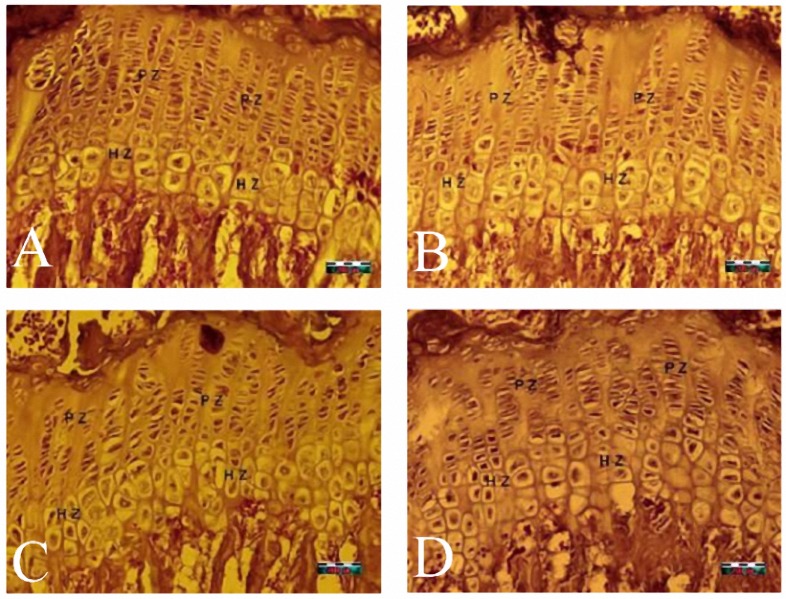
Images of the growth plate cartilage in different groups. The proliferation zone (PZ) and the hypertrophic zone (HZ) are marked. A: an image of non morphine-dependent rat cartilage after 4 weeks of follow-up. There is no evidence of acute/chronic inflammation, necrosis, fibrosis or hyalinisation. The chondrocytes are settled in the PZ in the form of regular and condensed columns. B: an image of non morphine-dependent rat cartilage after 7 weeks of follow-up. At this time point closer to the time of growth cessation, the accumulation of cells in the HZ has decreased. C: an image of morphine-dependent rat cartilage after 4 weeks of follow-up. D: an image of morphine-dependent rat cartilage after 7 weeks of follow-up. At this time point closer to the time of growth cessation, the accumulation of PZ cells has decreased. Original magnification 400×.

There were no signs of inflammation, fibrosis, necrosis or hyalinisation in the growth cartilages of different groups. Figure 3(A, B, C, D) shows four views from the growth plates of all groups at the aforementioned follow-up time points.

**Table 1.** Comparison of the cell number and growth plate thickness between morphine-dependent and non morphine-dependent groups and the duration of addiction (four weeks versus seven weeks). Data are shown as mean±SEM.

Groups	Non dependent		dependent		
Weeks	4	7	4	7	*P*
Cell number	185.0±1.1	187.7±0.85	177.5±1.2	164.6±1.7	0.000
Growth plate thickness (µ)	148.5±0.33	147.2±0.47	144.3±0.55	137.0±0.44	0.000

## Discussion

The findings of our study show that administration of morphine in male rats results in a significant decrease in the number of cells in the PZ and a significant decrease in the thickness of the growth plate, but does not influence the number of cells in the HZ of the growth plate (data are not shown). These findings indicate that morphine may exert an inhibitory effect on the proliferation of cells within the growth plate.

Hatsukari *et al* (2007) revealed that opioids have an effect on cell proliferation *in vitro *(1). During contact with morphine in cultured tumour cell lines, the hydroxyl group is replaced with a methoxy group, suggesting the involvement of a free radical-mediated reaction resulting in cell death. Therefore, using certain concentrations of morphine as an anti-nociceptive may also induce apoptosis or necrosis in proliferating cells. It has been suggested that morphine promotes apoptosis via the induction of *fas* and p53 gene expression. Also, Tegedar *et al* (2003) showed that inhibition of the *fas* gene results in the cessation of morphine-induced apoptosis (15). Induction of p53, an important apoptotic protein, is followed by DNA injury in the proliferation zone (16). 

Some studies have shown that different concentrations of morphine have a variable influence on cell proliferation. The results of a study by Singhal *et al* (1998) on kidney fibroblasts showed that low concentrations (physiologic; 10^-12 ^M) of morphine increase cell proliferation while higher concentrations (pharmacologic; 10^-8 ^M) cause apoptosis (17). Cell proliferation was mediated via the transference of the opioid receptor gene into the cell colony and also by chronic stimulation by morphine (18). This report has been confirmed by a study on the effect of morphine on hair follicle cell proliferation (19,20). As we used a pharmacologic dose of morphine (2×10^-3 ^M), we may suggest that apoptosis is involved in the reduction of the cell population in the PZ of the growth plate cartilage in morphine-dependent animals. 

Suppression of bone cell proliferation by morphine may cause a delay in the recovery process of bone fractures in morphine-dependent animals (9). Moreover, it has been shown that there are μ-opioid receptors on the chondrocytes of human joints and beta-endorphin binding sites have been observed on the chondrocytes of rat cartilage (2,21). The effect of morphine on cells through opioid receptors results in the inhibition of adenylate cyclase and a decrease in the amount of intracellular cAMP (21). The amount of intracellular cAMP depends on the amount of morphine (2, 17, 22-24). Taken together, these findings describe how morphine influences chondrocyte proliferation. Therefore, we can conclude that morphine is able to suppress cell proliferation, probably through binding to its specific receptors which are located on the surface of cartilage chondrocytes. 

In addition, Yilmaz *et al* (1999) found that the serum LH and testosterone levels decrease following the administration of morphine (8). Moreover, the use of narcotics (morphine and methadone) is consistent with inhibition of LH secretion and hypothalamus activity and a decrease in the level of serum testosterone in male rats (25). Testosterone administration leads to growth enhancement (26) through changes in DNA synthesis which result in increased chondrocyte proliferation (27). Therefore, in addition to the direct effects of morphine on cell proliferation, we can attribute our findings to a hormonal mechanism through which the hypothalamo-hypophyseal-gonadal axis was influenced. Hormonal alterations following morphine administration could lead to a decrease in serum testosterone levels and lower proliferation of chondrocytes in morphine-dependent animals.

It has been shown that administration of morphine reduces blood TSH levels, leading to a decrease in thyroid hormone release (28). Thyroid hormone can stimulate the differentiation process of hypertrophic zone chondrocytes and also inhibits chondrocyte proliferation (29). Data suggest that the primary target cells for T3 within the growth plate are progenitor chondroblasts, immature proliferating chondrocytes, and primary spongiosum osteoblasts, together with osteoblasts and osteocytes at adjacent sites of active bone turnover (20,26). Nevertheless, the results of Lewinson *et al* (1989) indicated that hypothyroidism could lead to a reduction in the width of the epiphyseal plate as a consequence of fewer proliferative cells and a reduction in the size of hypertrophic cells (30). Based on this contradictory evidence, whether we can attribute our results to alterations in TSH and thyroid hormones due to morphine administration is unclear. 

Regarding the fact that the reduction in the thickness of the growth cartilage was not accompanied by any change in the number of hypertrophic cells (data are not shown), we can probably attribute this reduction to the inhibition of cell proliferation in the PZ of the growth cartilage.

## Conclusion

In summary, the present study suggests that morphine reduces cell numbers in the proliferative zone and decreases the thickness of the growth plate of male rats, probably via its specific receptors on PZ chondrocytes or via a reduction in testosterone and thyroid hormone levels, resulting in reduced longitudinal growth of long bones. 

Although our study indicates some alterations in the growth plate cartilage subsequent to morphine administration, the precise mechanism of action is still unclear, and needs more investigation in terms of assessing apoptosis and hormonal changes after morphine administration. On the other hand, the effects of different doses of morphine on growth plate cartilage, especially after closure of the growth plate, and the study of changes in the length of long bones can provide new useful information. The results of this study showing growth plate cartilage alterations after morphine administration may alert clinicians in the use of prolonged morphine administration in young patients receiving analgesic drugs for long periods of time.
